# Pregnancy outcomes in women with gestational hypertension and preeclampsia at Paropakar Maternity and Women’s Hospital, Nepal: A retrospective study

**DOI:** 10.1371/journal.pone.0286287

**Published:** 2023-06-02

**Authors:** Seema Das, Renusha Maharjan, Rashmita Bajracharya, Rabina Shrestha, Sulata Karki, Rupesh Das, Jon Øyvind Odland, Maria Lisa Odland

**Affiliations:** 1 Research and Development Division, Department of Public Health and Community Programs, Dhulikhel Hospital Kathmandu University Hospital, Dhulikhel, Nepal; 2 Department of Sociology and Gerontology, Miami University, Oxford, Ohio, United States of America; 3 Department of Epidemiology and Public Health, School of Medicine, University of Maryland, Baltimore, Maryland, United States of America; 4 Department of Medicine, Janaki Medical College Teaching Hospital, Janakpur, Nepal; 5 Department of Public Health and Nursing, Norwegian University of Science and Technology, Trondheim, Norway; 6 School of Health Systems and Public Health, Faculty of Health Sciences, University of Pretoria, Pretoria, South Africa; 7 Department of Obstetrics and Gynecology, St Olav’s Hospital, Trondheim University Hospital, Trondheim, Norway; 8 Malawi-Liverpool-Wellcome Trust Research Institute, Blantyre, Malawi; 9 Institute of Life Course and Medical Sciences, University of Liverpool, Liverpool, United Kingdom; University of the Witwatersrand, SOUTH AFRICA

## Abstract

**Introduction:**

Gestational hypertension and preeclampsia are the most common types of hypertensive disorder in pregnancy and these conditions are associated with adverse maternal and fetal outcomes. This study aims to determine the differences in pregnancy outcomes in women with gestational hypertension and preeclampsia.

**Methods:**

A retrospective study was done at The Paropakar Maternity and Women’s Hospital, a tertiary level hospital, in the Kathmandu, Nepal. Pregnant women who had given birth at the hospital between September 17 and December 18 of 2017 were included. Data were obtained from the non-digitalized hospital records. The adjusted odds ratio (AOR) and 95% confidence interval were computed using logistic regression analysis. Multivariable analysis of pregnancy outcomes (cesarean sections, low birth weight, and preterm birth) was adjusted for maternal age, parity, twin birth, gestational age, calcium supplementation, and maternal co-morbidity.

**Results:**

Preeclampsia was strongly associated with cesarean section compared to normal pregnancies (OR = 8.11, p<0.001). Whereas the odds of cesarean section among women with gestational hypertension was almost 2 times (OR = 1.89, p<0.001). Preterm birth was not significantly associated with gestational hypertension but was associated with preeclampsia (OR = 3.39, p<0.001). Gestational hypertension and preeclampsia were not associated with low birth weight.

**Conclusion:**

In Nepal, women who develop preeclampsia seem at higher risk of having adverse pregnancy outcomes than women with gestational hypertension. These findings should be considered by national health authorities and other health organizations when setting new priorities to improve pregnancy outcomes.

## Introduction

Worldwide, about 10% of pregnancies are complicated with hypertensive disorders, and gestational hypertension and preeclampsia account for the majority of these cases [1]. Gestational hypertension (de novo) is defined as an increase in systolic blood pressure to 140 mm of Hg or higher and/or diastolic blood pressure to 90 mm of Hg or higher respectively on two consecutive readings (4 h apart) at ≥20 weeks of gestation in the absence of proteinuria or other findings suggestive of preeclampsia [2]. Preeclampsia (de novo) is defined as gestational hypertension accompanied by one or more of the following new-onset conditions at ≥20 weeks of gestation (i) proteinuria (24-h urine collection with a total protein excretion of 300mg or 1 + on urine dipstick); (ii) evidence of maternal organ dysfunction such as renal insufficiency (creatinine ≥ 90 umol/L), liver dysfunction (elevated transaminases with or without right upper quadrant or epigastric abdominal pain), neurological complications (eclampsia, altered mental status, blindness, stroke, severe headaches, or persistent visual scotomata), pulmonary oedema, hematological complications (e.g., platelet count <150, 000/uL; and (iii) uteroplacental dysfunction (such as placental abruption, angiogenic imbalance, fetal growth restriction, abnormal umbilical artery Doppler waveform, or intrauterine fetal death) [2]. Gestational hypertension is the most common complication which occurs in around 5%-10% of pregnancies, whilst preeclampsia affects about 2%-5% of pregnancies [3]. The prevalence of these disorders tends to be higher in lower and middle-income countries (LMIC) [4–6].

Both gestational hypertension and preeclampsia are associated with adverse maternal and fetal outcomes, including increased risk of future maternal cardiovascular diseases [7, 8], however complications due to gestational hypertension are less severe compared to preeclampsia [3]; still there is about 17% likelihood of progression of gestational hypertension towards preeclampsia [5].

Several studies have been conducted in developed and developing countries around the world to determine the maternal and perinatal outcomes associated with the hypertensive disorder [1, 6, 9–12]. However there are only a few studies [13, 14] that focus on pregnancy outcomes associated with these disorders in Nepal. Also, these studies did not compare the pregnancy outcomes of gestational hypertension and preeclampsia using adjusted statistical models [13, 14]. As such, the estimates from these [13, 14] studies does not account for confounding factors like maternal age and maternal comorbidity, which may lead to bias in risk estimates and thereby difficult identifying the magnitude of effects of gestational hypertension and preeclampsia on pregnancy outcomes. Addressing this gap will have important policy implications for developing strategies to improve pregnancy outcomes among women with gestational hypertension and preeclampsia in Nepal. Therefore, the aim of this study is to determine the difference in pregnancy outcomes in women with gestational hypertension and preeclampsia compared to women with normal pregnancies in one of the referral hospitals in capital city of Nepal.

## Materials and methods

The retrospective cross-sectional study was conducted at the Paropakar Maternity and Women’s Hospital, Kathmandu, Nepal. It is a tertiary level and first maternity hospital situated in Kathmandu district, Bagmati province, Nepal. About 22,000 women give birth in this hospital every year [15]. The study population (n = 4820) consisted of all the pregnant women who had given birth at the hospital between September 17 and December 18, 2017. We retrieved the data from the hospital’s official record files/patient charts. As the digitalized record was not available, all the available information about the mother and their newborn was entered into the study database using Microsoft Excel (Microsoft, NY, USA). A double data entry system was used to minimize the error.

### Measure

#### Outcome

The outcome variable of interest was pregnancy outcomes. Pregnancy outcomes included in this study were cesarean sections (both elective and emergency delivery), low birth weight (defined as babies who are born weighing less than 2500 grams), and preterm birth (defined as the birth of babies before 37 weeks gestation).

#### Exposure

The independent variables were gestational hypertension and preeclampsia. A two-step process was implemented to identify the women with gestational hypertension and preeclampsia. First, women with a recorded diagnosis of gestational hypertension and preeclampsia were identified. Second, we reviewed patient’s charts for specific clinical and laboratory findings and compared them with World Health Organization (WHO) criteria. According to WHO, gestational hypertension is defined as systolic and diastolic blood pressure to ≥140 mm of Hg and ≥90 mm of Hg, respectively in two or more consecutive occasions (≥4 h apart) after 20 weeks of gestation without proteinuria [16]. Similarly, WHO defined preeclampsia as an increase in systolic and diastolic blood pressure to ≥140 mm of Hg and ≥90 mm of Hg respectively in two or more consecutive occasions (≥4 h apart) after 20 weeks of gestation with proteinuria > 0.3 g/24 hour or ≥ 1 measured by a urine dipstick [16].

#### Covariates

Maternal age, parity, type of pregnancy, previous cesarean section, and maternal co-morbidity potential confounders were selected on the basis of existing literature and biologic plausibility [3]. Maternal age at the time of delivery was categorized as ≤ 30 years and ≥ 30 years. Parity was defined as the number of previous live births and stillbirths and was dichotomized into primiparity and multiparity. The type of pregnancies was designated as singleton and twin pregnancy. Previous cesarean section and history of preeclampsia were defined on the basis of recorded maternal history. Maternal co-morbidities included in the study were: Chronic hypertension (recorded diagnosis); gestational diabetes mellitus (defined as the increased blood sugar level after 20 weeks of gestational age, and fasting glucose level ≥ 6.7 mmol/L; urinary tract infection (recorded diagnosis and white blood cells in urine sample and/or urine culture report); hypothyroidism (recorded diagnosis and/or abnormal thyroid function test report); and asthma and sub-fertility treatment (recorded diagnosis). Participants who were affected with at least one of these diseases were included in maternal co-morbidity.

#### Statistical analysis

The information obtained was cleaned, sorted, and coded to facilitate data analysis. The statistical analysis was done using Statistical Package for Social Science (SPSS) version 24 (IBM, NY, USA). Frequency and percentage of maternal characteristics and pregnancy outcomes were estimated for normal pregnancies (woman without gestational hypertension and preeclampsia), gestational hypertension and preeclampsia and whether these distributions were significant was evaluated by using the chi-square test and fisher’s exact test, when appropriate.

Logistic regression analysis was used to compare pregnancy outcomes in participants affected with gestational hypertension and preeclampsia with normal pregnancies (woman without gestational hypertension and preeclampsia).

Separate models were created for women with only gestational hypertension, women with only preeclampsia and women with normal pregnancies (as the reference) in each model.

Each model was adjusted for maternal age, parity, twin births, gestational age, calcium supplementation, previous cesarean section, history of preeclampsia, and maternal co-morbidity. Initially, univariable models were conducted. All the variables included in this preliminary step were also included in the subsequent multivariable analysis and the forced entry method was applied. Although the variables were not significantly associated with the pregnancy outcome, we still included them in the multivariable model because they are possible confounders based on the existing literature. Multi-collinearity was examined using variance inflation factor (VIF). Final models were tested for overall goodness-of-fit using the Hosmer-Lemeshow test. The results are presented as Crude odds ratio (COR), adjusted odds ratio (AOR), and their 95% Confidence intervals (CIs). The cut-off value for the level of significance was set as ≤ 0.05. A descriptive analysis was done to make sure that each category has less than 5% of missing data [17]. Woman with both gestational hypertension and preeclampsia were excluded from the analysis.

### Ethical consideration

Ethical approval was obtained from Nepal Health Research Council and a permission letter was obtained from the research committee of the Paropakar Maternity and Women’s Hospital. Due to the retrospective study design, informed consents were not obtained, and all the collected data were analyzed anonymously. Study participants or the public were not involved in the design, conduct, reporting, or dissemination plans of our research.

## Results

Out of the total sample size of 4820, the incidence of preeclampsia was 85 (1.8%) and gestational hypertension was 205 (4.3%) among pregnant women admitted to Paropakar and Women’s Maternity Hospital. Four women with both gestational hypertension and preeclampsia were excluded making the final sample available for analysis, 4816.

Table 1 shows the distribution of maternal characteristics in pregnant women with normal pregnancies, gestational hypertension, and preeclampsia. Compared to women who had a normal pregnancy (14.2%), preeclampsia (28.4%) and gestational hypertension (20.9%) were more common among pregnant women over the age of 30 years (p = <0.001). Regarding maternal comorbidity, and iron and calcium supplementation, the distribution of women with gestational hypertension and preeclampsia was significantly different (p = <0.001). For maternal co-morbidity, almost 19% of those with preeclampsia had at least one maternal co-morbidity. In contrast, the proportions were comparatively low for gestational hypertension and normal pregnancies at about 2%. Calcium supplementation was given to nearly 99% of women with normal pregnancies and gestational hypertension, and only 90% of women with preeclampsia (<0.001).

**Table 1:** Distribution of maternal characteristics in pregnant women with normal pregnancies, gestational hypertension and preeclampsia.

	Normal pregnancies	Gestational hypertension	Preeclampsia	
Maternal Characteristics	n (%)	n (%)	n (%)	p-value
**Maternal age**				
≤30	3888 (85.8)	159 (79.1)	58 (71.6)	**<0.001**
>30	646 (14.2)	42 (20.9)	23 (28.4)	
**Parity**				
Primiparity	2545 (56.1)	116 (57.7)	53 (65.4)	0.228
Multiparity	1989 (43.9)	85 (42.3)	28 (34.6)	
* **Calcium Supplementation**				
No	45 (1.0)	3 (1.5)	8 (9.9)	**<0.001**
Yes	4324 (99.0)	194 (98.5)	73 (90.1)	
**Pregnancies**				
Singleton	4506 (99.4)	200 (99.5)	76 (93.8)	**<0.001**
Twin birth	28 (0.6)	1 (0.5)	5 (6.2)	
**Previous Cesarean section**				
No	4321 (95.3)	189 (94.0)	78 (96.3)	0.642
Yes	213 (4.7)	12 (6.0)	3 (3.7)	
**Maternal co-morbidity** †				
No	4438 (97.9)	197 (98.0)	66 (81.5)	**<0.001**
Yes	96 (2.1)	4 (2.0)	15 (18.5)	

^†^Maternal co-morbidity = Chronic Hypertension, Urinary Tract Infection, Hypothyroidism, Gestational Diabetes, Sub-fertility treatment, and Asthma.

*Missing data n = 169

Note: Four women with both gestational hypertension and preeclampsia were excluded from analysis

The distribution of pregnancy outcomes among women with normal pregnancies, gestational hypertension and preeclampsia is presented in Table 2. The pregnancy outcomes of interest were the type of delivery, birth weight, and pre-term birth. A higher proportion of women with preeclampsia (72.8%) and women with gestational hypertension (39.3%) had cesarean sections compared to normal pregnancies (25.5%) (p = <0.001). About 90% of newborns had a normal birth weight (>2500 g) in all three categories of pregnant women. In women with preeclampsia 33.3% had a preterm birth which was significantly higher than gestational hypertension (9.0%) and normal pregnancies (9.9%) (p = <0.001).

**Table 2:** Distribution of pregnancy outcomes of women with normal pregnancies, gestational hypertension and preeclampsia.

	Normal pregnancies	Gestational hypertension	Preeclampsia	
Pregnancy outcomes	n (%)	n (%)	n (%)	p-value
**Type of delivery**				
Normal	3377 (74.5)	122 (60.7)	22 (27.2)	**<0.001**
Cesarean	1157 (25.5)	79 (39.3)	59 (72.8)	
**Birth weight** *				
<2500	4059 (90.0)	174 (88.8)	72 (88.9)	0.809
≥2500	450 (10.0)	22 (11.2)	9 (11.1)	
**Preterm Birth**				
No	4085 (90.1)	183 (91.0)	54 (66.7)	**<0.001**
Yes	449 (9.9)	18 (9.0)	27 (33.3)	

* Missing data n = 30

Note: Four women with both gestational hypertension and preeclampsia excluded from analysis

Crude odds ratio with 95% confidence interval of pregnancy outcomes of gestational hypertension and preeclampsia can be found in Table 3. The odds of cesarean section among women with gestational hypertension were higher than the women with normal pregnancies (OR = 1.89, p = <0.001). Likewise, women with preeclampsia were more likely to have cesarean section than women with normal pregnancies (OR = 7.83, p = <0.001). The odds of low birth weight among women with gestational hypertension and women with preeclampsia were about 14% and 13% higher (respectively) to that of women with normal pregnancies; however, the relationships were not statistically significant. Furthermore, there was no significant association between gestational hypertension and preterm birth (p = 0.65). However, the odds of preterm birth among women with preeclampsia were higher than the odds of the women with normal pregnancies (OR = 4.55, p = <0.001).

**Table 3:** Crude odds ratio with 95% confidence interval of pregnancy outcomes of gestational hypertension and preeclampsia.

	Cesarean section		Low birth weight		Preterm Birth	
	Crude OR [95% CI]	p-value	Crude OR [95% CI]	p-value	Crude OR [95% CI]	p-value
**Gestational hypertension (Reference-Normal pregnancies)**	1.89 [1.41–2.52]	**<0.001**	1.14 [0.72–1.79]	0.56	0.89 [0.54–1.46]	0.65
**Preeclampsia (Reference-Normal pregnancies)**	7.83 [4.77–12.83]	**<0.001**	1.13 [0.56–2.27]	0.72	4.55 [2.83–7.29]	**<0.001**

Note: OR refers to odds ratio; CI refers to confidence interval; woman with both gestational hypertension and preeclampsia were excluded in this model

Table 4 presents the results of a multivariable analysis assessing pregnancy outcomes of gestational hypertension and preeclampsia. All adjusted odds were somewhat lower than crude odds. Women with gestational hypertension were 89% more likely to have cesarean section than that of women with normal pregnancies (AOR = 1.89, p = <0.001), controlling for maternal age, parity, twin birth, calcium supplementation, previous cesarean section, history of preeclampsia, and maternal co-morbidity. Similarly, compared to women with normal pregnancies, women with preeclampsia were more likely to have cesarean section (AOR = 8.11, p<0.001). Gestational hypertension was not significantly associated with low birth weight (AOR = 1.04, p = 0.87) and preterm birth (AOR = 0.89, p = 0.63) in the present study. Also, there was no significant relationship between preeclampsia and low birth weight (AOR = 1.14, p = 0.72). However, the odds of preterm births were higher among women with preeclampsia than that of women with normal pregnancies (AOR = 3.39, p = <0.001), controlling for all other variables in the model.

**Table 4:** Adjusted odds ratio with 95% confidence interval of pregnancy outcomes of gestational hypertension and preeclampsia.

	Cesarean section^a^		Low birth weight^b^		Preterm Birth^a^	
	Adjusted OR [95% CI]	p-value	Adjusted OR [95% CI]	p-value	Adjusted OR [95% CI]	p-value
**Gestational hypertension (Reference-Normal pregnancies)**	1.89 [1.38–2.59]	**<0.001**	1.04 [0.65–1.67]	0.87	0.89 [0.54–1.46]	0.63
**Preeclampsia (Reference-Normal pregnancies)**	8.11 [4.81–13.66]	**<0.001**	1.14 [0.56–2.34]	0.72	3.39 [2.04–5.62]	**<0.001**

^a^ adjusted for maternal age, parity, twin births, calcium supplementation, previous cesarean section, history of preeclampsia and maternal co-morbidity.

^b^ adjusted for maternal age, parity, twin birth, gestational age, calcium supplementation, previous cesarean section, history of preeclampsia and maternal co-morbidity.

Note: Woman with both gestational hypertension and preeclampsia were excluded in this model

## Discussion

In this study, we found the pregnancy outcomes such as cesarean section, low birth weight and preterm birth were common among women with preeclampsia and gestational hypertension. Both crude odds ratio and adjusted odds ratio depicted the adverse effects on pregnancy outcomes in women with gestational hypertension and preeclampsia compared to women with normal pregnancies. However, gestational hypertension and preeclampsia showed some differences with the occurrence of adverse effects on pregnancy outcomes. Adverse pregnancy outcomes were more prevalent among the women with preeclampsia compared to gestation hypertension including increased odds of delivery by cesarean sections and preterm birth.

Most of the women were under 30 years of age for gestational hypertension and preeclampsia. The finding is slightly different in a similar study conducted among women with preeclampsia in Finland [18]. However, our finding is in line with the study conducted among women with gestational hypertension and preeclampsia in Nepal [14]. Advanced maternal age, twin births, and maternal co-morbidity varied significantly across the three groups of pregnant women whilst women with preeclampsia had higher age, twin births, and more comorbidities. This is similar to have been reported in few studies [3, 6]. These discrepancies with other studies might be due to ethnicity, cultural and geographical factors, and other confounding factors that were not controlled in our study.

Gestational hypertension and preeclampsia were associated with delivery type, and preeclampsia with preterm birth. Birth weight however was not statistically significant with gestational hypertension and preeclampsia in this study. Our study showed that a higher proportion of women with preeclampsia had a cesarean section. This was expected and similar to what other studies on severe preeclampsia and hypertensive disorders have found [19, 20]. In both women with gestational hypertension and women with preeclampsia in this study, around 12% of the newborn had low birth weight (<2500 g). This is somewhat lower than what has been found previously in a systematic review, which reported a pooled prevalence of low birth weight of 37% in women with hypertensive disorders of pregnancy [21]. However, a cohort study showed a higher incidence of preterm birth among the women with preeclampsia [3] which is in line with our findings.

Similar to a previous study we found that both gestational hypertension and preeclampsia are associated with cesarean section and the odds of cesarean section among women with preeclampsia are significantly higher than that of gestational hypertension [3]. A higher risk of cesarean section in the preeclampsia group compared to gestational hypertension might be because preeclampsia requires a shorter time to develop complications affecting both mother and fetus [3] and the definitive treatment is to end the pregnancy to ensure the safety of both the mother and fetus [22]. Many patients and obstetricians prefer cesarean section over vaginal delivery because cesarean section expedites delivery which might reduce the risk of morbidity and perinatal death [23]. However, studies have supported and recommended vaginal delivery in preeclampsia if there is no other indication of cesarean section [19, 24]; even so health professionals fear that vaginal delivery will worsen the maternal and fetal condition [19, 23]. Further, our study was not able to categorize preeclampsia with or without severe features; as preeclampsia with severe features might lead to more cesarean deliveries [6]. Hence, further research is required to find the reason for increased number of cesarean deliveries in preeclampsia cases in Nepal.

Our study didn’t find a significant association between gestational hypertension and preeclampsia with a low birth weight compared to normotensive women. This is interesting as previous studies’ findings on this have been inconsistent [25, 26]. A retrospective study conducted in Canada found no significant difference in the birth weight of full-term newborns delivered by women with preeclampsia, gestational hypertension, and normotensive women at term birth [25]. Similarly, a study conducted in an urban sub-Saharan African setting also showed the same findings [27]. On the other hand in a different study in a low resources setting in Ghana, women with preeclampsia had a higher risk of low birth weight than normotensive women adjusted for gestational age, maternal age, parity, type of delivery, and education [7]. A reason for not observing a higher risk of low birth weight among women with gestational hypertension and preeclampsia is that we did not control for some of the confounders such as maternal body mass index, maternal nutrition, and other socio-demographic factors. More research is required to explore the association of low birth weight with gestational hypertension and preeclampsia by controlling these confounders.

Several studies have [7, 12, 21, 26] shown that both gestational hypertension and preeclampsia are associated with preterm birth. However, gestational hypertension increases the risk of preterm birth with a much smaller effect size [3]. On the other hand, our study reported a significant association of preeclampsia with an increased risk of preterm birth, while gestational hypertension was not significantly associated with preterm birth. This might be due to the effects of confounders (body mass index, ethnicity, residence area, and socioeconomic status), type of study design, and study population diversity. Similarly, there was a statistically significant association between the severity of the hypertensive disorder and preterm birth [6]. Hence, it will be easier to manage less severe hypertensive disorders and prevent complications like preterm birth if the frequency of antenatal visits increases as regular follow-up and adequate antenatal screening are important for timely management and prevention of complications from hypertensive disorders in pregnancy [28]. Gestational hypertension is less severe preeclampsia field [6] as preeclampsia is associated with vascular manifestations, oxidative stress and endothelial damage leading to poor placental function [27]. Poor placental function affects the perfusion and nutrients supplementation to the fetus, which can result in preterm birth [27]. Similarly, iatrogenic preterm birth by early termination of pregnancy could also be the reason for preterm birth [29].

### Strengths and limitations

This is the first known study conducted at Paropakar Maternity and Women’s Hospital in Nepal to estimate the risk of gestational hypertension and preeclampsia on pregnancy outcomes by performing multivariable regression analysis. As gestational hypertension and preeclampsia are both associated with adverse maternal and fetal conditions, it is important to shed light on the severity of these two spectrums. This study tried to compare the severity of pregnancy outcomes between gestational hypertension and preeclampsia. Therefore, this study serves as a reference for further study as it provides the baseline data on perinatal outcomes associated with gestational hypertension and preeclampsia.

Our study was limited to only one tertiary-level hospital in Nepal. Although most of the babies born in an institutional setting are delivered at the study hospital, the national percentage of institutional deliveries is only 63% [30]. Therefore, the findings may not be generalizable to the national population of pregnant women due to the large number of women who never come to the hospital for check-ups and gave home births. Likewise, limiting our study to only a single hospital may have caused unintended selection bias. Moreover, the non-digitalized nature of the data may have led to some data being lost. There is an increased possibility of clerical errors, as our data were extracted from the hospital’s official paper-based patient records and charts which could have led to information bias and the chance of misclassification. Many potential confounders were not controlled for due to a lack of pertinent information, for instance, socio-demographic data such as ethnicity, level of education, occupational status, residence area, smoking status; and maternal factors such as maternal obesity, gestational weight gain, type 1, and type 2 diabetes mellitus; and fetal factors such as intrauterine growth retardation, macrosomia, and Apgar scores. This might have introduced bias in our estimate.

## Conclusion

Adverse pregnancy outcomes were more prevalent among the women with preeclampsia including increased risk of cesarean sections and preterm birth. This indicates that women who developed preeclampsia are potentially at higher risk of adverse pregnancy outcomes than women with gestational hypertension. Therefore, the findings from this study will be valuable for national health authorities and other health organizations when setting new priorities to improve pregnancy outcomes. Antenatal consultation should be more focused on early recognition of hypertensive disorders, better management and referral to higher centers which might help to prevent complications in pregnancies and improve maternal and newborn outcomes. This can be achieved through training and orientation programs for all health professionals involved in maternal and child health care at all levels in primary health care and hospital. Also, these findings should alert policymakers to the increased rate of cesarean deliveries in preeclampsia cases. However, more research is required to understand the impact of hypertensive disorder in pregnancy in other clinical settings in Nepal such as primary and secondary healthcare facilities.

## Supporting information

S1 Dataset (XLSX)
